# Telomerase reverse transcriptase coordinates with the epithelial-to-mesenchymal transition through a feedback loop to define properties of breast cancer stem cells

**DOI:** 10.1242/bio.034181

**Published:** 2018-06-15

**Authors:** Ahmed El-Badawy, Nehal I. Ghoneim, Mohamed A. Nasr, Hoda Elkhenany, Toka A. Ahmed, Sara M. Ahmed, Nagwa El-Badri

**Affiliations:** 1 Center of Excellence for Stem Cells and Regenerative Medicine (CESC), Zewail City of Science and Technology, 6th of October City 12588, Egypt; 2 Department of Surgery, College of Veterinary Medicine, Alexandria University, Alexandria 22785, Egypt

**Keywords:** HTERT, CSCs, Cancer metastasis, Chemoresistance

## Abstract

Telomerase and its core component, telomerase reverse transcriptase (hTERT), are critical for stem cell compartment integrity. Normal adult stem cells have the longest telomeres in a given tissue, a property mediated by high hTERT expression and high telomerase enzymatic activity. In contrast, cancer stem cells (CSCs) have short telomeres despite high expression of hTERT, indicating that the role of hTERT in CSCs is not limited to telomere elongation and/or maintenance. The function of hTERT in CSCs remains poorly understood. Here, we knocked down hTERT expression in CSCs and observed a morphological shift to a more epithelial phenotype, suggesting a role for hTERT in the epithelial-to-mesenchymal transition (EMT) of CSCs. Therefore, in this study, we systematically explored the relationship between hTERT and EMT and identified a reciprocal, bi-directional feedback loop between hTERT and EMT in CSCs. We found that hTERT expression is mutually exclusive to the mesenchymal phenotype and that, reciprocally, loss of the mesenchymal phenotype represses hTERT expression. We also showed that hTERT plays a critical role in the expression of key CSC markers and nuclear β-catenin localization, increases the percentage of cells with side-population properties, and upregulates the CD133 expression. hTERT also promotes chemoresistance properties, tumorsphere formation and other important functional CSC properties. Subsequently, hTERT knockdown leads to the loss of the above advantages, indicating a loss of CSC properties. Our findings suggest that targeting hTERT might improve CSCs elimination by transitioning them from the aggressive mesenchymal state to a more steady epithelial state, thereby preventing cancer progression.

## Abstract (precis)

**Summary:** This study describe a reciprocal, bi-directional feedback loop between hTERT and EMT to regulate properties of CSCs, suggesting that targeting hTERT may eliminate CSCs, thereby preventing cancer progression.

## INTRODUCTION

Breast cancer remains a challenging medical problem worldwide and is the most frequently diagnosed cancer and the most common invasive cancer in women (McGuire et al., 2015). Strategies targeting primary breast tumors have markedly improved; however, the poor prognosis of patients with advanced breast cancer is primarily because of the high frequency of tumor metastasis (Redig and McAllister, 2013). Metastasis is a complex process that ultimately causes cancer-related death (Gupta and Massagué, 2006). Cancer cell metastasis is now known to occur through the acquisition of an invasive mesenchymal phenotype, a process called the epithelial-to-mesenchymal transition (EMT) (Heerboth et al., 2015). The acquisition of mesenchymal traits promotes motility and invasiveness in malignant cells. Furthermore, cancer cell EMT is associated with amplified cell stemness and resistance to treatment (Gupta et al., 2009; Mani et al., 2008). The hallmarks of EMT includes E-cadherin downregulation, which is essential for cell-cell adhesion, and N-cadherin upregulation, which marks the mesenchymal phenotype (Huber et al., 2005).

Cancer stem cells (CSCs) are currently considered the driving force of cancer progression and metastasis because of their tumor initiation properties and resistance to chemotherapeutic agents (El-Badawy et al., 2017; Salem et al., 2015). Therefore, understanding the cellular and molecular mechanisms that regulate CSCs will be essential for the development of CSC-targeted therapies. Human telomerase reverse transcriptase (hTERT) is an RNA-dependent DNA polymerase that synthesizes telomeric DNA at chromosomal ends to maintain telomere length (Liu et al., 2010). hTERT is absent in most human somatic cells due to transcriptional repression soon after embryogenesis (Liu et al., 2010). In contrast, hTERT activity is relatively high in tissue stem and progenitor cells (Shay and Wright, 2007). Interestingly, up to 90% of human malignancies are associated with high hTERT expression and telomerase activation, which are positively correlated with tumor aggressiveness (Shay and Bacchetti, 1997). Additionally, hTERT has been shown to be involved in critical oncogenic pathways (Koh et al., 2015; Li et al., 2016). Accordingly, it was proposed that targeting telomerase or telomere structure might be an effective therapy for cancer (Harley, 2008). Although a cell has only few molecules of TERT (Akincilar et al., 2015), hTERT has also been reported to be actively involved in many processes such as cell signaling, proliferation, apoptosis, and migration. (Chang and DePinho, 2002; Cong and Shay, 2008). However, the role of hTERT in the functional properties of CSCs remains unclear. Moreover, although recent studies have reported a link between hTERT and EMT in different cancer models (Liu et al., 2013; Qin et al., 2016), it remains unclear whether this association also affects CSC properties. Accordingly, we explored the functional importance of hTERT and the link between hTERT and EMT in CSCs using breast CSCs as a model. Here, we report a bi-directional link in which hTERT and EMT reciprocally affect each other and coordinate bilaterally to regulate the functional properties of CSCs from MDA-MB-231 cells.

## RESULTS

### hTERT expression regulates the epithelial-to-mesenchymal transition in breast CSCs

**Fig. 1.**
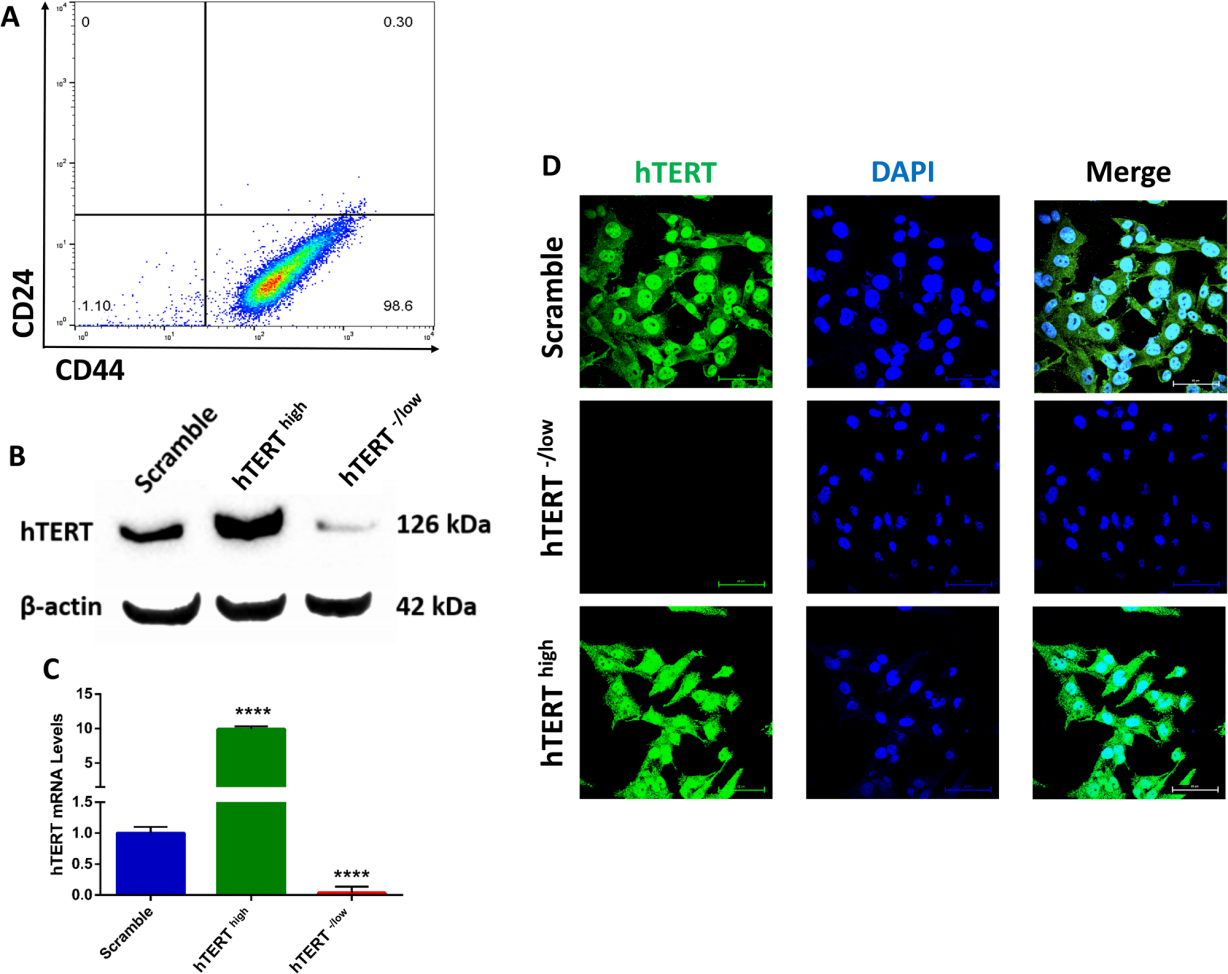
**Verification of hTERT knockdown and overexpression in breast CSCs.** (A) Flow cytometry plot for cell surface markers CD44 and CD24 in sorted CSCs confirming pure population. Gating is set to an isotype control. (B) Western blot analysis confirming hTERT knockdown and upregulation compared with control scrambled cells. β-actin was used to ensure the loading of equal amounts of protein. (C) Real-time qRT-PCR analysis of hTERT mRNA expression confirming downregulation and upregulation compared with control scrambled cells. β-Actin mRNA was used to normalize the variability in template loading. The data are reported as the means±s.d. (D) Confocal immunofluorescence images for hTERT (green) confirming hTERT knockdown. Nuclei were stained with DAPI (blue). Scale bars: 60 µM.

**Fig. 2.**
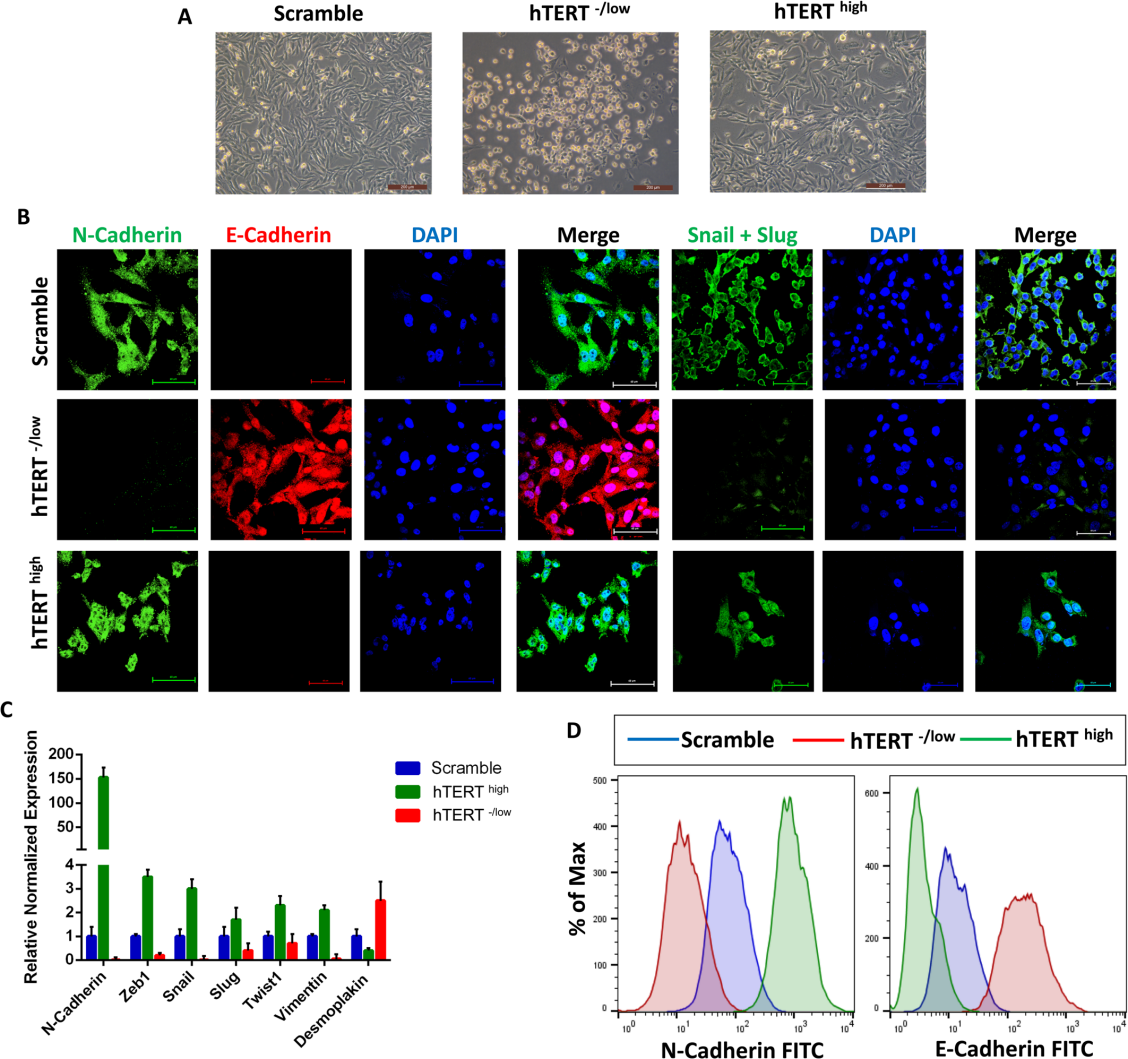
**hTERT plays a critical role regulating the epithelial-to-mesenchymal transition in CSCs.** (A) Phase-contrast images showing hTERT^-/low^ CSCs have an epithelial phenotype, whereas hTERT^high^ CSCs have a mesenchymal phenotype. (B) Confocal immunofluorescence images for N-cadherin (green), E-cadherin (red) and Snail+Slug (green) in control CSCs, CSCs overexpressing hTERT and hTERT-knockdown CSCs. Nuclei were stained with DAPI (blue). Scale bars: 60 µM. (C) The expression levels of mRNAs encoding N-cadherin, Zeb1, Snail, Slug, Twist, Vimentin and Desmoplakin in CSCs overexpressing hTERT and hTERT-knockdown CSCs relative to control CSCs as determined by real-time qRT-PCR. The data are reported as the means±s.d. (D) Flow cytometry overlay histogram analysis of N-cadherin and E-cadherin in control CSCs and CSCs overexpressing hTERT and hTERT-knockdown CSCs. For comparison, an isotype control was used to define the positive and negative population for each marker.

To investigate the importance and function of hTERT in CSCs, we sorted CD44^+^CD24^−^ breast CSCs and confirmed higher than 98% purity (Fig. 1A). Next, we upregulated or downregulated hTERT expression in CD44^+^CD24^−^ breast CSCs. Cells expressing a scrambled shRNA were used as controls. We first confirmed hTERT overexpression or knockdown in CD44^+^CD24^−^ breast CSCs by western blotting, immunofluorescence and qPCR (Fig. 1B–D). Cells overexpressing hTERT were fibroblast-like and showed mesenchymal morphology, whereas cells with hTERT knockdown exhibited an epithelial morphology (Fig. 2A). This result led us to analyze a panel of epithelial and mesenchymal markers in hTERT-overexpressing and knockdown CSCs. Confocal immunofluorescence analysis for the expression of various transcription factors (EMT-TFs) known to control the EMT process indicated that the expression of the epithelial marker E-cadherin was downregulated in hTERT^high^ CSCs, whereas E-cadherin was upregulated in hTERT^−/low^ CSCs. By contrast, the expression of mesenchymal markers (such as N-cadherin, Snail and Slug) was upregulated in hTERT^high^ CSCs and downregulated in hTERT^−/low^ CSCs. (Fig. 2B). Further characterization by real-time qPCR revealed significantly higher levels of mesenchymal marker mRNAs in hTERT^high^ CSCs, whereas hTERT^-/low^ CSCs showed significantly decreased mesenchymal marker expression and increased epithelial marker expression (Fig. 2C). In addition, flow cytometry analysis confirmed that hTERT^high^ CSCs were mesenchymal, as shown by N-cadherin overexpression, and hTERT^-/low^ CSCs were epithelial, as shown by E-cadherin overexpression (Fig. 2D).

### hTERT expression changes to reflect the epithelial or mesenchymal state of breast CSCs

**Fig. 3.**
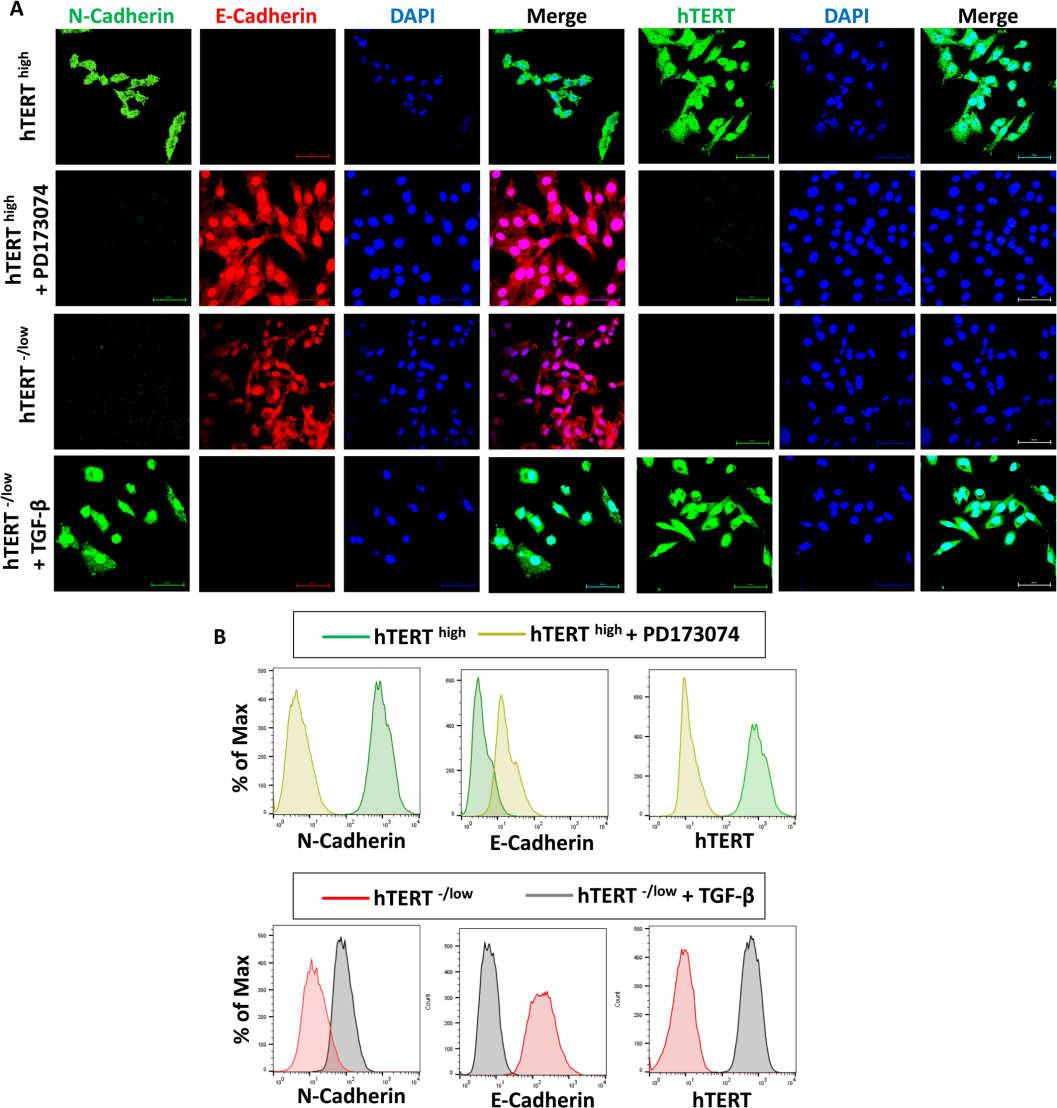
**hTERT expression in CSCs is mutually exclusive with the mesenchymal phenotype.** (A) Confocal immunofluorescence images for N-cadherin (green), E-cadherin (red) and Snail+Slug (green) showing that the loss of mesenchymal phenotype in hTERT^high^ CSCs mediated by PD173074 is associated with the loss of hTERT expression. However, acquisition of a mesenchymal phenotype in hTERT^-/low^ CSCs mediated by TGF-β is associated with increased hTERT expression. Nuclei were stained with DAPI (blue). Scale bars: 60 µM. (B) Flow cytometry overlay histogram analysis of N-cadherin, E-cadherin and hTERT showing that hTERT^high^ CSCs treated with PD173074 lose their mesenchymal phenotype, which was associated with a loss of hTERT expression. Additionally, hTERT^-/low^ CSCs treated with TGF-β acquired a mesenchymal phenotype, which was associated with increased hTERT expression. For comparison, an isotype control was used to define the positive and negative population for each marker.

Because we showed that hTERT affects the EMT in CSCs, we asked whether this effect was reciprocal or whether the epithelial or mesenchymal state affects hTERT expression in breast CSCs. To this end, we treated mesenchymal hTERT^high^ CSCs with PD173074 for 4 days, which is known to induce the mesenchymal-to-epithelial transition (Nguyen et al., 2013). We confirmed that mesenchymal hTERT^high^ CSCs transitioned into an epithelial state by assessing E-cadherin and N-cadherin expression. This transition in hTERT^high^ CSCs from a mesenchymal to an epithelial state was associated with a loss of hTERT expression (Fig. 3A). Similarly, we treated epithelial hTERT^-/low^ CSCs with TGF-β for 4 days, which is a potent activator of the mesenchymal state (Asiedu et al., 2011; Gregory et al., 2011; Katsuno et al., 2013). We confirmed that epithelial hTERT^-/low^ CSCs transitioned into a mesenchymal state by assessing N-cadherin and E-cadherin expression. This transition in hTERT^-/low^ CSCs from an epithelial to a mesenchymal state was associated with an increase in hTERT expression (Fig. 3A). Furthermore, flow cytometry analysis confirmed that the change from a mesenchymal state to an epithelial state in hTERT^high^ CSCs by PD173074 was associated with decreased hTERT expression, and the change from an epithelial state to a mesenchymal state in hTERT^-/low^ CSCs by TGF-β was associated with increased hTERT expression (Fig. 3B). These data suggest reciprocal regulation between the mesenchymal state and hTERT expression.

### hTERT regulates CSC properties

**Fig. 4.**
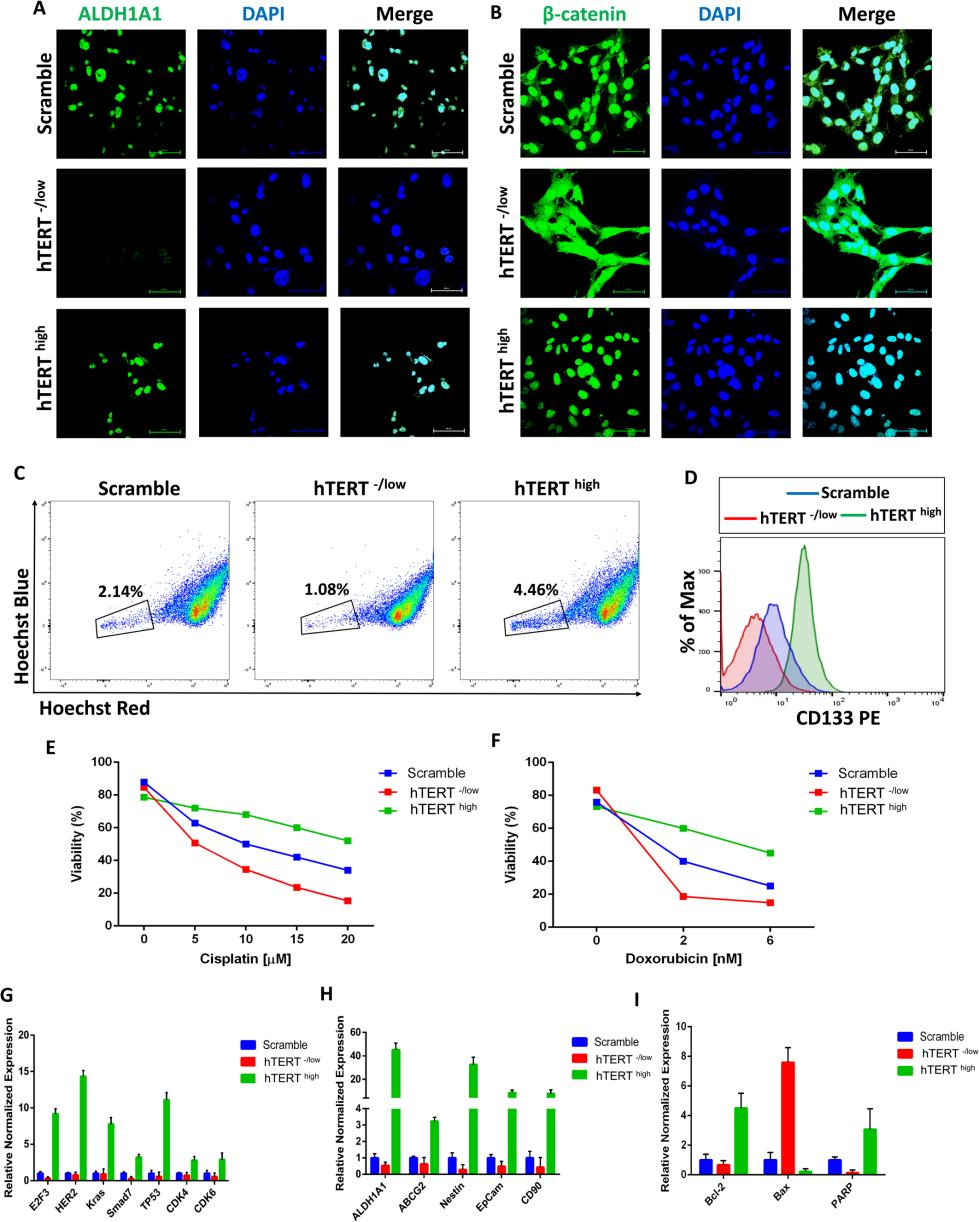
**hTERT defines CSC properties.** (A) Confocal immunofluorescence images for ALDH1A1 (green) showing that hTERT^high^ CSCs are positive for ALDH1A1, whereas hTERT^-/low^ CSCs do not express ALDH1A1. Nuclei were stained with DAPI (blue). Scale bars: 60 µM. (B) Confocal immunofluorescence images for β-catenin (green) showing cytoplasmic localization of β-catenin in hTERT^-/low^ CSCs, whereas hTERT^high^ CSCs showed nuclear localization of β-catenin. Nuclei were stained with DAPI (blue). Scale bars: 60 µM. (C) Side population (SP) analysis by flow cytometry indicating more SP cells in hTERT^high^ CSCs than in hTERT^-/low^ CSCs, which have fewer SP cells than do control CSCs. (D) Flow cytometry analysis of CD133 showing that hTERT^high^ CSCs have higher CD133 expression than do control CSCs, whereas hTERT^-/low^ CSCs are negative for CD133 expression. An isotype control was used to define the positive and negative populations. (E–F) hTERT^high^ CSCs, hTERT^-/low^ CSCs and control CSCs were exposed to increasing concentrations of cisplatin (E) or doxorubicin (F) for 24 h. Cell viability was determined by Annexin-V-FITC and PI apoptosis detection kits. hTERT^high^ CSCs showed more significant resistance to cisplatin and doxorubicin than did control CSCs, whereas hTERT^-/low^ CSCs exhibited a relative loss of chemoresistance capabilities (*P*<0.05). (G) The expression levels of the cancerous markers E2F3, HER2, KRas, SMAD7, TP53, CDK4 and CDK6 are higher in hTERT^high^ CSCs as determined by real-time qRT-PCR. The data are reported as the means±s.d. (H) Real-time qRT-PCR analysis of CSC marker genes showing higher expression levels in hTERT^high^ CSCs. β-actin mRNA was used to normalize variability in template loading. The data are reported as the means±s.d. (I) Real-time qRT-PCR analysis of Bcl-2 (an anti-apoptotic protein) and Bax (a pro-apoptotic molecule indicating a significantly increased expression of Bcl-2 and reduced expression of Bax in hTERT^high^ CSCs compared with that in control CSCs (*P*<0.05). β-actin mRNA was used to normalize the variability in template loading. The data are reported as the means±s.d.

We next examined the functional role of hTERT on CSC properties. Aldehyde dehydrogenase 1A1 (ALDH1A1) has been shown to be a potential stemness marker and plays a role in CSC biology (Fleischman, 2012; Moreb, 2008). Additionally, it plays a crucial role in chemoresistance pathways, and its levels correlate with disease prognosis (Ginestier et al., 2007; Huang et al., 2013). Examination of ALDH1A1 expression in hTERT^high^ and hTERT^-/low^ CSCs at the protein level by immunofluorescence staining showed that hTERT^high^ CSCs exhibit high ALDH1A1 expression (Fig. 4A), whereas hTERT^-/low^ CSCs were negative for ALDH1A1 expression (Fig. 4A). This result suggests a critical role for hTERT in the expression of the CSC marker ALDH1A1.

To identify the possible molecular pathway(s) enabling the observed effect of hTERT on breast CSCs, we analyzed Wnt/β-catenin signaling in hTERT^high^ and hTERT^-/low^ CSCs. The Wnt/β-catenin signaling pathway is essential for CSC function (Eaves and Humphries, 2010; Espada et al., 2009; Nusse, 2008; Reya and Clevers, 2005). For instance, mammary stem cells with high levels of Wnt/β-catenin signaling have greater tumorigenic potential than their counterparts with low Wnt/β-catenin signaling levels (Monteiro et al., 2014). Moreover, Wnt/β-catenin signaling regulates CSC self-renewal, tumorigenesis and cancer chemoresistance (Mohammed et al., 2016). Moreover, the nuclear accumulation of β-catenin is correlated with CSC properties, and malignant cells with nuclear-localized β-catenin are especially abundant in the invasive front of many cancers (Fodde and Brabletz, 2007; Li and Zhou, 2011). Our data showed that control CSCs have both nuclear and cytoplasmic β-catenin as shown by immunofluorescence confocal imaging (Fig. 4B). However, β-catenin in hTERT^-/low^ CSCs was cytoplasmic, whereas hTERT^high^ CSCs showed nuclear β-catenin (Fig. 4B), suggesting aberrant activation of β-catenin in hTERT^high^ CSCs, which indicates that hTERT functions by activating the β-catenin pathway.

CSCs exclude Hoechst 33342 dye (and chemotherapy drugs) because they express multidrug-resistant transporters such as ABCG2, known as side population (SP) cells (Challen and Little, 2006; Moserle et al., 2010). The SP assay has been widely used as an indicator of stemness, and SP cells have been proven to be enriched in CSCs from thyroid cancer (Mitsutake et al., 2007), ovarian cancer (Szotek et al., 2006), breast cancer (Patrawala et al., 2005), glioma (Kondo et al., 2004), melanoma (Dou et al., 2009) and hepatocellular carcinoma (Chiba et al., 2006). Thus, we compared hTERT^high^ and hTERT^-/low^ CSCs for Hoechst dye exclusion as an indicator of SP properties. hTERT^-/low^ CSCs had a lower SP cell percentage than control CSCs (Fig. 4C). However, hTERT^high^ CSCs exhibited a high percentage of SP cells (Fig. 4C), indicating a role of hTERT in SP properties. Additionally, hTERT^high^ CSCs showed higher expression of CD133, a marker used to identify CSCs (Wu and Wu, 2009), than hTERT^-/low^ CSCs (Fig. 4D).

CSCs have been reported to be relatively resistant to chemotherapy (Dean et al., 2005). Because hTERT influenced CSC marker expression and exhibited an effect on SP properties, we investigated the effect of changing hTERT expression in response to conventional chemotherapeutic agents using the Annexin-V-FITC and PI apoptosis detection kit, which are markers of apoptosis. hTERT^high^ and hTERT^-/low^ CSCs were exposed for 24 h to varying concentrations of cisplatin and doxorubicin, anticancer chemotherapeutic medications. hTERT^high^ CSCs were more resistant than control and hTERT^-/low^ CSCs to two commonly used chemotherapeutic drugs, cisplatin (Fig. 4E) and doxorubicin (Fig. 4F). Chemotherapy-induced cell death was significantly increased in hTERT^-/low^ CSCs relative to control cells, indicating a critical role for hTERT in breast CSC chemoresistance. In response to cisplatin and doxorubicin treatment, hTERT^high^ CSCs expressed significantly less Annexin-V than control and hTERT^-/low^ CSCs (data not shown), indicating that hTERT affects apoptotic resistance. To investigate the possible mechanism enabling hTERT to block chemotherapy-induced apoptosis in CSCs, we used qPCR to analyze the expression of Bcl-2 (an anti-apoptotic protein) and Bax (a pro-apoptotic molecule). Bcl-2 was overexpressed, whereas the pro-apoptotic molecule Bax was downregulated in hTERT^high^ CSCs (Fig. 4G), suggesting that hTERT blocks chemotherapy-induced apoptosis in breast CSCs by preferential activation of the Bcl-2 cell survival response. These findings can be further supported by previous reports that hTERT regulates NF-κB signaling, a well know anti-apoptotic factor (Ghosh et al., 2012). Compared with control and hTERT^-/low^ CSCs, qPCR analysis showed that hTERT^high^ CSCs overexpress poly-ADP-ribose polymerase (PARP), which plays an essential role in DNA repair (Fig. 4G). Overall, these data show that hTERT plays a critical role in the resistance of breast CSCs to chemotherapeutic agents, which as previously discussed (Li and Tergaonkar, 2014; Ozturk et al., 2017), can provide a potential therapeutic targeting for CSC based therapies.

To assess the effect of hTERT on the expression of cancer related genes in breast CSCs, the expression of previously reported candidate cancer genes in hTERT^high^ CSCs was compared with control and hTERT^-/low^ CSCs. qPCR for E2F3, HER2, KRAS, SMAD7, TP53, CDK4 and CDK6, which are associated with the acquisition of a cancerous phenotype, showed higher expression in hTERT^high^ CSCs (Fig. 4H). qPCR analysis showed increased expression of many CSC marker genes (Keysar and Jimeno, 2010; Klonisch et al., 2008; Medema, 2013) such as ALDH1, ABCG2, NESTIN, EpCam and CD90, altogether suggesting that hTERT plays a significant role in the expression of cancer and CSC markers.

### hTERT enhances migration, tumorsphere formation and colony formation:

**Fig. 5.**
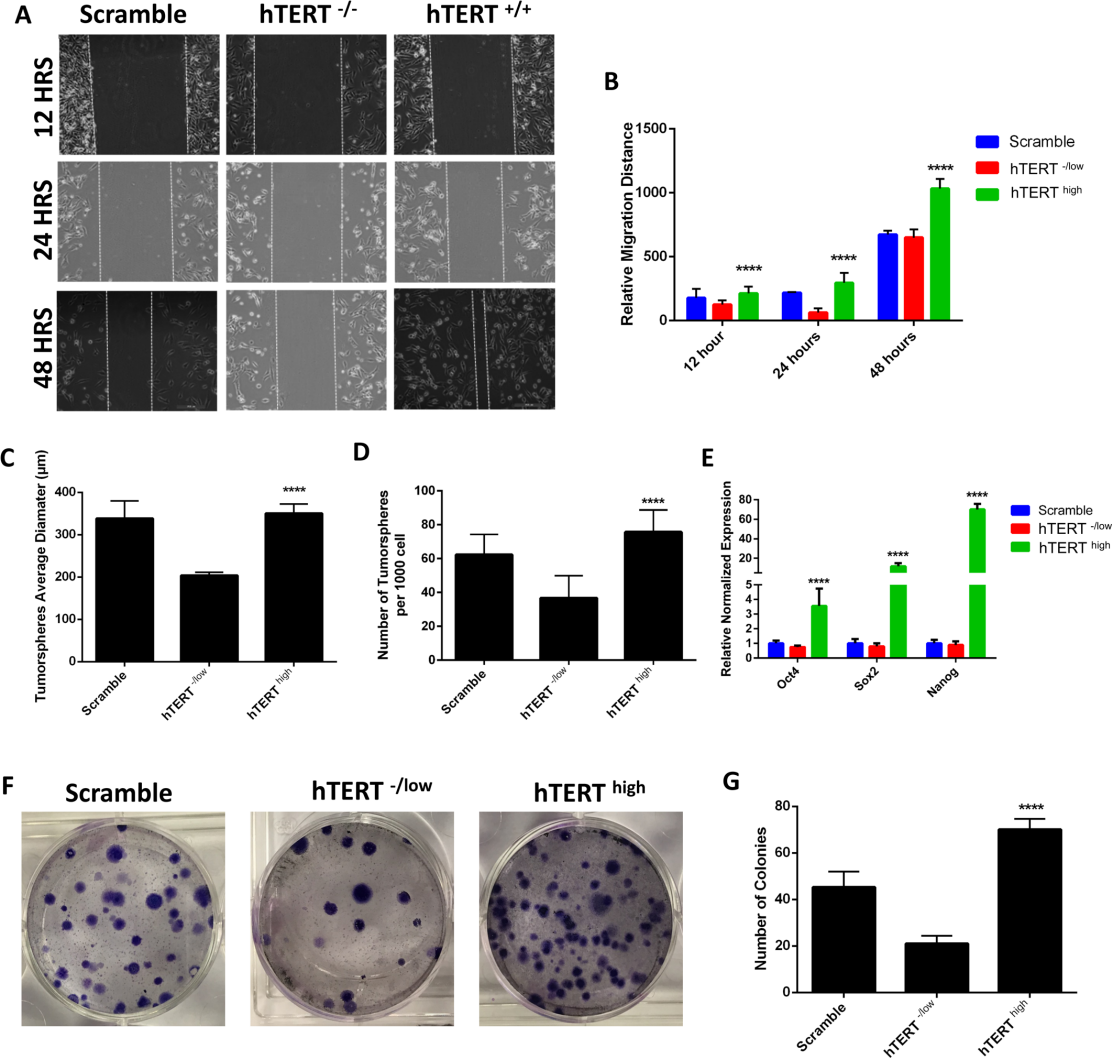
**Role of hTERT in migration, tumorsphere and colony formation of CSCs.** (A) Scratch wound healing assay indicating that hTERT^high^ CSCs have higher migration capacities than hTERT^-/low^ CSCs and control CSCs. (B) Relative migration distance of hTERT^high^ CSCs, hTERT^-/low^ CSCs and control CSCs, related to A. (C–D) Quantification of tumorsphere-forming ability of hTERT^high^ CSCs, hTERT^-/low^ CSCs and control CSCs showing that hTERT^high^ CSCs have significantly higher tumorsphere formation percentages as shown by average tumorsphere size (C) and number (D). The data are represented as the means±s.d. (****P<0.05). (E) The expression levels of the pluripotency markers OCT4, SOX2 and NANOG are higher in hTERT^high^ CSCs than in control CSCs as determined by real-time qRT-PCR. The data are reported as the means±s.d. (****P<0.05). (F) Images showing the colony formation capacities of hTERT^high^ CSCs, hTERT^-/low^ CSCs and control CSCs and (G) quantification of the number of colonies formed. hTERT^high^ CSCs to have higher colony formation capabilities. Data are represented as the means±s.d. (****P<0.05).

We investigated the effect of hTERT on the migration capacity of CSCs, a critical factor involved in metastasis (Balic et al., 2006; Hermann et al., 2007). A scratch wound healing assay was used to quantitatively evaluate cell migration. As shown in Fig. 5A and B, hTERT^high^ CSCs exhibited higher migration capacities than control cells, whereas hTERT^-/low^ CSCs showed decreased migration. We next examined the role of hTERT in CSC self-renewal capacity by assessing tumorsphere-forming ability in suspension culture, an *in vitro* measure of stem cell activity (Dontu et al., 2003). hTERT^high^ CSCs showed significantly higher tumorsphere-forming ability than control cells, whereas hTERT^-/low^ CSCs formed fewer tumorspheres (Fig. 5C,D). Because of hTERT's observed significance in tumorsphere formation, an indicator of self-renewal capacity, we investigated the effect of hTERT on the expression of pluripotency markers. We found that cells overexpressing hTERT expressed significantly higher levels of pluripotency markers than control or hTERT^-/low^ CSCs (Fig. 5E).

A critical feature of stem cells is their capacity to self-renew and generate hierarchically organized structures in which their progeny loses their self-renewal capacity during differentiation (Clevers, 2011; Salem et al., 2015). Thus, we assayed the role of hTERT in the capacity of CSCs to generate many progeny by the colony formation assay, which can determine the functional heterogeneity among cancer cells derived from the brain, lung and ovary tumors (Franken et al., 2006; Hamburger and Salmon, 1977). We initiated a series of clonogenic experiments to determine the colony formation capacities of hTERT^high^ and hTERT^-/low^ CSCs. hTERT^high^ CSCs showed a higher capacity to form colonies and produce large numbers of progeny than control and hTERT^-/low^ CSCs, indicating a role for hTERT in the self-renewal and tumorigenic potential of CSCs (Fig. 5F,G).

### hTERT improves resistance to stress-induced injury and enhances proangiogenic activities in breast CSCs

**Fig. 6.**
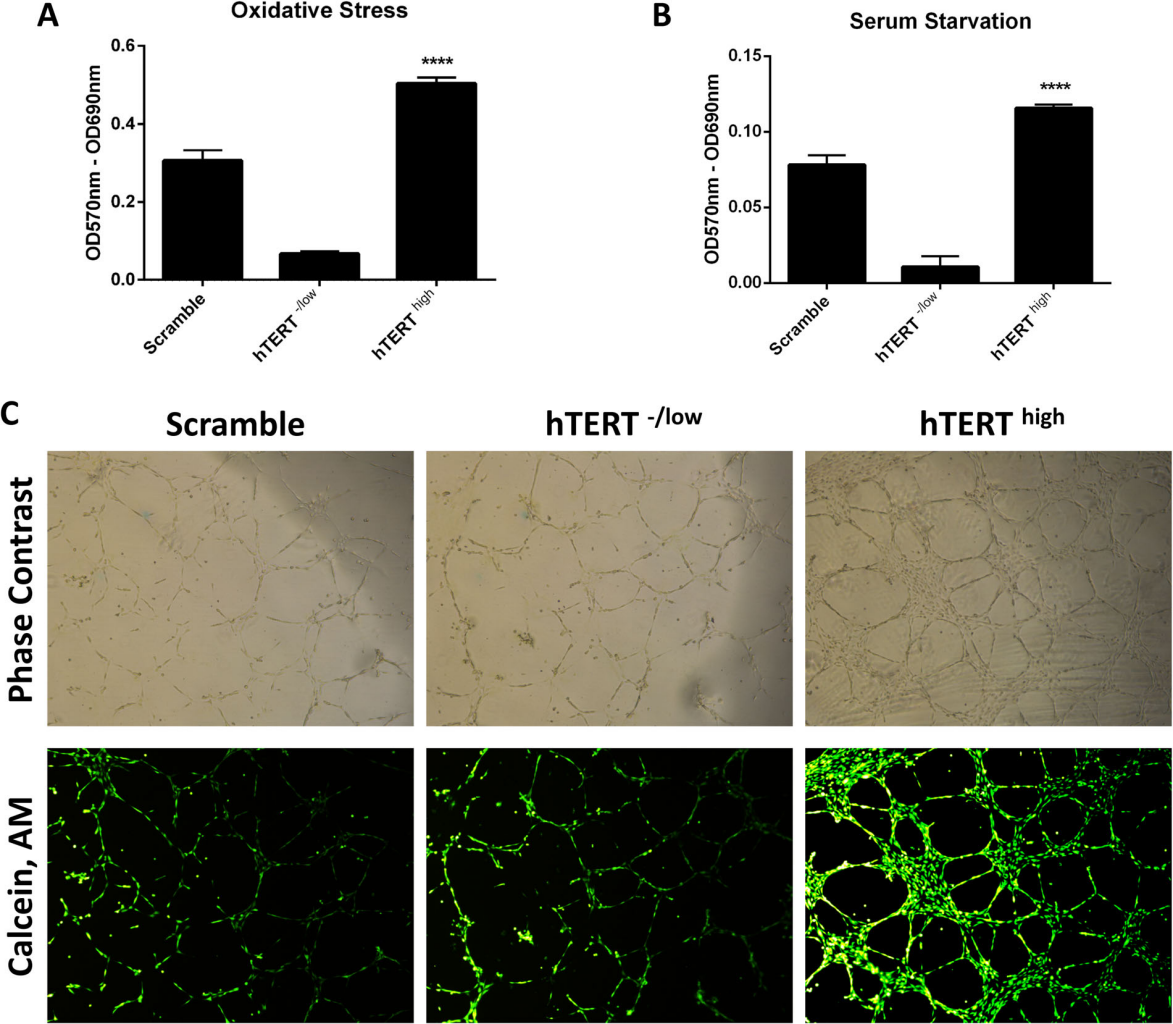
**hTERT plays a vital role in CSC resistance to stress-induced injury and contributes to the pro-angiogenic properties of CSCs.** (A–B) MTT assay (5 mg/ml) to evaluate the viability rates of hTERT^high^ CSCs, hTERT^-/low^ CSCs and control CSCs after exposure to (A) oxidative stress or (B) serum starvation. hTERT^high^ CSCs are more resistant to stress-induced injury. Formazan absorbance at 570 nm with reference to 630 nm expressed as a measure of cell proliferation (****P<0.05). (C) The upper panel is a phase-contrast image of the field shown on the below panel. Representative images of the tubular structures from the *in vitro* tube formation assay were photographed and showed hTERT^high^ CSCs to have higher vascularization capacities. Scale bars: 500 μm.

The tumor microenvironment and cancer cells are often exposed to intrinsic and extrinsic stress, such as oxidative stress and nutrient starvation, which can stimulate tumor aggressiveness (Osawa et al., 2013). More importantly, the induction of oxidative stress and nutrient starvation is one of the underlying mechanisms of action for many anticancer drugs and radiation. Because CSCs are known to resist therapy, we investigated the role of hTERT in the resistance to oxidative stress injury and nutrient starvation. The MTT results showed hTERT^high^ CSCs to be more resistant to both oxidative stress-induced injury and nutrient starvation than control cells, and hTERT^-/low^ CSCs displayed increased sensitivity to stress and starvation injury (Fig. 6A,B). These data suggest that hTERT helps cells adopt a system to counteract oxidative stress-induced injuries and nutrient starvation in CSCs, which might provide clues regarding how CSCs evade therapies that induce oxidative stress and nutrient starvation.

CSCs have been shown to play roles other than tumor initiation and the local regrowth of cancers following treatment and/or in the development of metastases. For example, CSCs have been shown to differentiate into endothelial cells, playing an important role in supporting tumor vascularization (Ricci-Vitiani et al., 2010). Following this line of reasoning, we examined the role of hTERT in the CSC vascularization process using an *in vitro* tube formation assay. hTERT^high^ CSCs displayed higher vascularization potentials as assessed by increased formation of more extensive networks of hollow, capillary tube-like structures than control cells and hTERT^-/low^ CSCs (Fig. 6C). This result suggests a role for hTERT in the CSC vascularization potential.

### Assessment of hTERT and its link to EMT in clinical cases of invasive breast cancer

As described above, we found a critical role for hTERT in breast CSCs and the maintenance of the CSC state. We also found an important reciprocal link between hTERT expression and EMT. Indeed, this link contributes to enhanced tumor initiation and progression. We were interested in relating these observations to the properties of clinical invasive breast cancer cases. To pursue this question, we accessed data from the Cancer Genome Atlas Network (Cancer Genome Atlas, 2012).

**Fig. 7.**
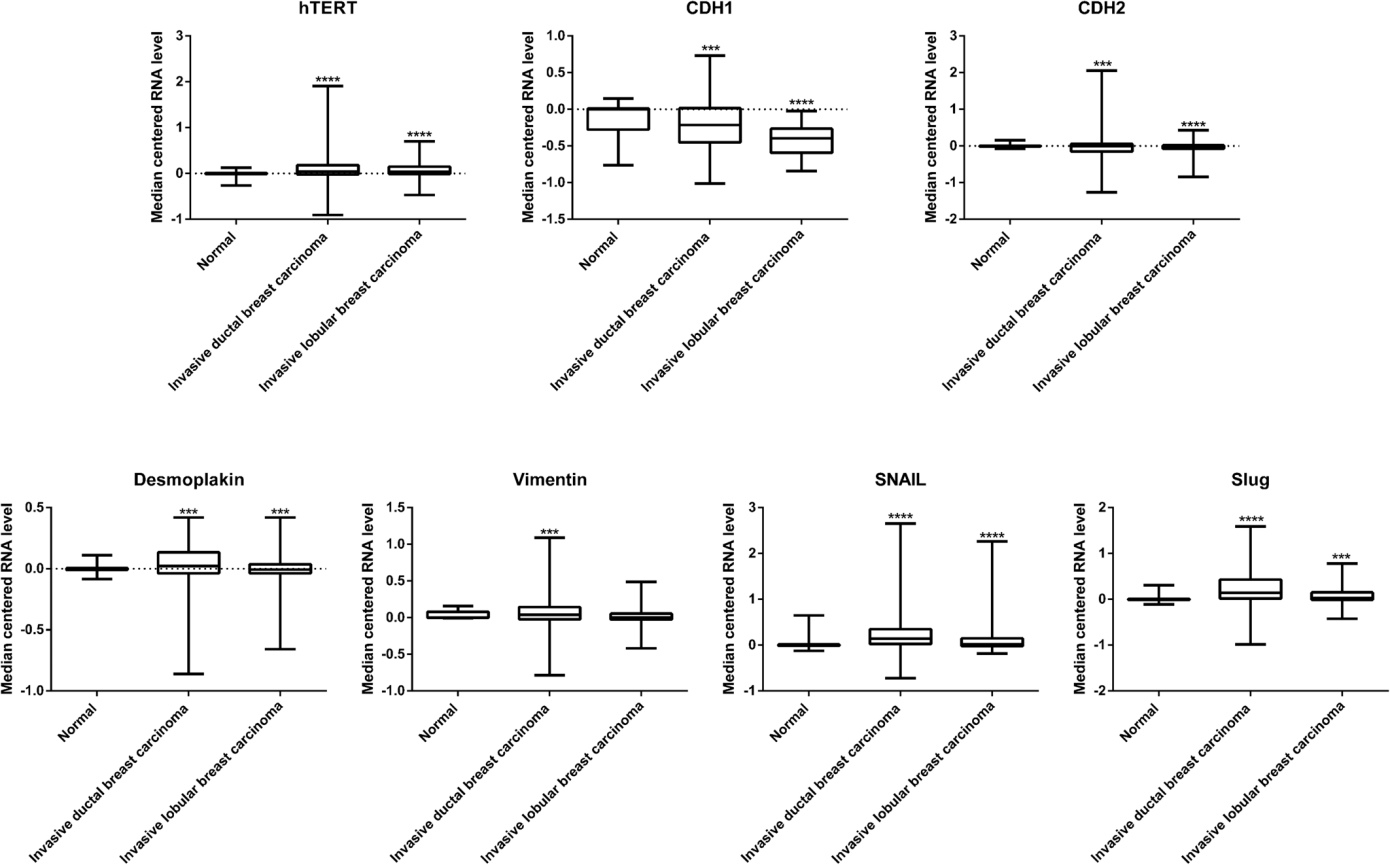
**Correlation of hTERT and EMT in clinical cases of breast cancer.** mRNA abundance of hTERT, E-cadherin, N-cadherin, Desmoplakin, Vimentin, Snail and Slug in clinical cases of invasive ductal and lobular breast carcinomas. Asterisks indicate a difference compared with the control normal patients. ***P<0.05; ****P<0.01.

First, we accessed the relative abundance of TERT expression and found a significant increase in the expression of hTERT levels in invasive forms of ductal (*P*-value<0.0001, 95% CI -0.2633029 to -0.1457771) and lobular (*P*-value<0.0001, 95% CI -0.1278589 to -0.0558411) breast carcinomas compared with that in normal tissue (Fig. 7). We next analyzed the expression levels of important markers regulating the EMT process and found decreased expression of E-cadherin in invasive forms of ductal (*P*-value=0.002, 95% CI 0.024810 to 0.111190) and lobular (*P*-value<0.0001, 95% CI 0.215719 to 0.363481) breast carcinomas and decreased expression of Desmoplakin in invasive forms of ductal (*P*-value=0.0024, 95% CI 0.01501722 to 0.06983078) and lobular (*P*-value=0.0021, 95% CI 0.01442823 to 0.06527977) breast carcinomas.

We next analyzed the expression of mesenchymal markers and found increased expression of N-cadherin in the invasive forms of ductal (*P*-value=0.0123, 95% CI -0.1544321 to -0.0187079) and lobular (*P*-value<0.0001, 95% CI 0.0538636 to 0.1155764) breast carcinomas. The Snail transcriptional factor also showed increased expression in the invasive forms of ductal (*P*-value<0.0001, 95% CI -0.3908573 to -0.2402227) and lobular (*P*-value<0.0001, 95% CI -0.3347394 to -0.1663406) breast carcinomas. The Slug transcriptional factor showed a similar increase in the invasive forms of ductal (*P*-value<0.0001, 95% CI -0.2395837 to -0.1332963) and lobular (*P*-value=0.0033, 95% CI -0.0978884 to -0.0195316) breast carcinomas. Vimentin also showed an increase in the invasive forms of ductal (*P*-value=0.0238, 95% CI -0.0816653 to -0.0057547) and lobular (*P*-value=0.1457, 95% CI -0.0060373 to 0.0406173) breast carcinomas. Taken together, we observed decreased expression of epithelial markers (E-cadherin and Desmoplakin) and found increased expression of mesenchymal markers (N-cadherin, Zeb1, Snail, Slug and Vimentin) along with an increased expression of hTERT in invasive breast cancers (Fig. 7).

Together with our demonstration of a reciprocal link between hTERT expression and EMT in breast CSCs, the high expression of hTERT in invasive breast cancers raises the possibility that the more aggressive nature of hTERT-high cancers may be, in part, attributable to the ability of hTERT to activate a mesenchymal status, which in turn might contribute to tumor progression, metastasis and therapy resistance.

## DISCUSSION

hTERT has been shown to be highly expressed in most human cancers (Harley, 2008). The induction of hTERT expression and telomerase activation are prerequisites for malignant transformation and cellular immortalization (Hanahan and Weinberg, 2000). However, the mechanism of hTERT involvement in cancer progression remains incompletely understood. In addition to the role for hTERT in maintaining telomere length in cancer cells, previous studies have shed light on the multiple biological functions of hTERT during carcinogenesis independent of telomere-based activity (Li and Tergaonkar, 2014). For example, hTERT can induce the expression of vascular endothelial growth factor (Kirkpatrick et al., 2004), and cells overexpressing hTERT are more resistant to different insults, including chemotherapeutic treatments (Dudognon et al., 2004; Zhang et al., 2017). Furthermore, the overexpression of hTERT in normal stem cells can enhance their mobilization and proliferation, which is achieved by activation of the canonical Wnt pathway (Park et al., 2009). TERT's non-canonical functions can act through different mechanisms by directly binding to promoters and transcriptional factors such as NF-κB (Ghosh et al., 2012) and Myc (Koh et al., 2015), or by regulating translation (Khattar et al., 2016). The mechanism of reactivation of telomerase in cancers has been recently reported to take place by recruitment of transcription factor such as GABPA (Akincilar et al., 2016) or BRAF (Li et al., 2016) specifically to mutant TERT promoters, hence driving TERT transcription.

Telomerase and its core component hTERT are critical for stem cell compartment integrity (El-Badawy and El-Badri, 2015). Normal adult stem cells are known to have the longest telomeres in a given tissue, which is mediated by the upregulation of hTERT (Flores et al., 2006; Hiyama and Hiyama, 2007). In CSCs, short telomeres have been reported from breast (Ponti et al., 2005), brain (Marian et al., 2010a), prostate (Marian et al., 2010b), myeloma (Brennan and Matsui, 2009) and leukemia (Cleary, 2009) tissue. Although they express high levels of telomerase and hTERT, CSCs do not appear to use this high hTERT expression for elongation and/or maintenance of telomere length. Thus, a better understanding of the molecular importance of hTERT in CSCs will help refine approaches to target telomerase in CSCs. Previous studies have showed that the biology of CSCs is tightly linked with the EMT process (May et al. 2011; Morel et al., 2008). Here, we report a critical role for hTERT in CSCs and show reciprocal bi-directional coordination between hTERT and EMT to define CSC properties. We show that hTERT expression in CSCs is associated with a mesenchymal phenotype and that loss of the mesenchymal phenotype and acquisition of an epithelial state are associated with the loss of hTERT expression. We also demonstrated a critical role for hTERT in the functional properties of breast CSCs.

Our experiments showed that hTERT expression in CSCs was positively regulated by acquiring a mesenchymal phenotype, implying the involvement of hTERT in inducing the EMT process in CSCs. We propose a double-positive feedback loop between hTERT and the mesenchymal phenotype of CSCs, in which the expression of hTERT is mutually exclusive to the mesenchymal phenotype and loss of the mesenchymal phenotype represses hTERT expression. By this logic, we predict that triggering of the EMT process in CSCs is determined by hTERT expression. Additionally, regarding the demonstrated role of hTERT in the EMT of CSCs, we also identified a key role for hTERT in the functional properties of CSCs. We showed that hTERT is important for the expression of key CSC markers, promoted the nuclear localization of β-catenin, increased the percentage of cells with SP properties and upregulated CD133 expression. Overexpression of hTERT in CSCs enhanced the chemoresistance properties of CSCs and upregulated cancer marker expression. hTERT also has functions in the migratory properties of CSCs and enhanced colony and tumorsphere formation, indicating a role for hTERT in CSC self-renewal. hTERT overexpression in CSCs enhanced survival under stressful conditions and enhanced vasculogenic activity. Subsequently, hTERT knockdown led to the loss of all the above properties, indicating a loss of CSC properties.

In summary, we identified a reciprocal feedback mechanism controlling hTERT and EMT in breast CSCs that sheds new light on the control of EMT in CSCs. Currently, many successful therapies designed to directly inhibit telomerase (Chiappori et al., 2015) or hTERT activity (Lü et al., 2012) exist. Our results provide clues to the mode of action behind the success of telomerase-based therapies, which occurs through an ability to inhibit CSC activity. Overall, targeting hTERT might help eliminate CSCs by transitioning them from the aggressive mesenchymal state to a steady epithelial state, thereby preventing cancer progression. We believe that future clinical trials designed to evaluate the efficacy of telomerase inhibitors on CSCs will indeed aid in developing approaches that target tumors from their root.

## MATERIALS AND METHODS

### Cells, breast CSC isolation and culture

MDA-MB-231 breast cancer cells (ATCC, Manassas, VA, USA) were maintained in DMEM supplemented with 10% fetal bovine serum (FBS), streptomycin, and penicillin (Life Technologies) at 37°C in a humidified incubator containing 5% CO_2_. No further authentication was performed for the cell line. For isolation of breast CSCs from MDA-MB-231, cells were washed once with PBS and trypsinized with 0.05% trypsin/EDTA. After centrifugation, cells were re-suspended in PBS containing 1% FBS (wash buffer), and stained with the following monoclonal antibodies for 30 minutes: FITC anti-CD44, PE anti-CD24. The respective isotype control for each marker was used to define the positive and negative population. CD44^+^CD24^−^ cells, which is a characteristic phenotype for breast CSCs (Horimoto et al., 2016; Jaggupilli and Elkord, 2012), were sorted by flow cytometry and cultured in clonogenic numbers in CSC medium consisting of DMEM/F12 medium (Life Technologies) with 2% B27 supplement (Life Technologies), 20 ng/ml epidermal growth factor (EGF, Life Technologies), 20 ng/ml basic fibroblast growth factor (FGF-b, Life Technologies) and 10 µg/ml insulin (Sigma-Aldrich). The purity of sorted cells was analyzed using FACSCalibur (Becton Dickinson, New Jersey, USA) following standard procedures using CellQuest Pro Software (Becton Dickinson). The clone with the highest purity was used for further experiments.

### Plasmids, transfections and clone selection

CD44^+^CD24^−^ CSCs were transfected with either pMKO.1 puro hTERT shRNA (Addgene, plasmid 10688) or pBabe-puro hTERT (Addgene, plasmid 1771) to knockdown or overexpress hTERT, respectively, in CD44^+^CD24^-^ CSCs. Cells transfected with a scramble shRNA (Addgene, plasmid 1864) served as control. In brief, each plasmid was co-transfected with packaging plasmids pCMV-VSV-G (Addgene, plasmid 8454) and pCL-Eco (Addgene, plasmid 12371) into virus packaging cell line HEK 293T (ATCC) using FuGENE HD Transfection Reagent (Promega, Lyon, France) following the standard procedure. The culture medium was changed after 24 h with fresh DMEM medium supplemented with 10% FBS. The conditioned medium containing viruses was collected in the following two consecutive days and polybrene (8 μg/ml) was added into the virus-containing medium. Then the culture media of the candidate CD44^+^CD24^−^ CSCs were replaced with the lentivirus-containing media. After 24 h, the virus-infected cells were selected with puromycin (1 μg/ml) and cultured in the presence of puromycin for 3 weeks to generate clones of stable cell lines of hTERT^high^ and hTERT^-/low^ CD44^+^CD24^−^ CSCs. These cells were collected and used for subsequent experiments.

### Flow Cytometry characterization

For flow cytometry analysis, cells were first incubated in a blocking solution (PBS containing 1% BSA) for 10 min and then centrifuged. For extracellular staining, cells were re-suspended in the blocking solution mixed with the following monoclonal antibodies: FITC-conjugated anti-CD44, PE-conjugated anti-CD24 and FITC-conjugated anti-CD133 and incubated for 30 min at 4°C in the dark. For intracellular staining, cells were fixed with 4% paraformaldehyde, permeabilized with 0.1% Triton X-100, and blocked with 4% BSA. The cells were then stained with hTERT antibody (Abcam), E-Cadherin antibody (Cell Signaling Technology), N-Cadherin antibody (Abcam), Snail+Slug antibody (Abcam), ALDH1A1 antibody (Pierce Antibodies, Waltham, MA, USA) and β-Catenin antibody (Cell Signaling Technology). Cells were then labeled with the appropriate Alexa Fluor^®^ secondary antibodies (Molecular Probes, Eugene, OR, USA). Flow cytometry was carried out using FACSCalibur (Becton Dickinson) following standard procedures using CellQuest Pro Software (Becton Dickinson). Data analysis was performed using FlowJo v. 10.2 software (Treestar, Ashland, OR, USA) with super-enhanced Dmax (SED) subtraction analysis for determination of differences in histograms.

### Side-population (SP) assay

Cells were trypsinized from tissue culture plates, suspended in prewarmed DMEM containing 2% FBS, and stained with 5 µg/ml of Hoechst 33342 dye (Molecular Probes) for 90 min at 37°C. Cells were then washed and resuspended in HBSS containing 2% FBS. Immediately before flow cytometry analysis, 2 µg/ml propidium iodide (Sigma-Aldrich) was added to exclude dead cells. SP cells were identified using flow cytometry after Hoechst dye excitation with a 350 nm UV laser.

### Cell lysis, SDS-PAGE and western blotting

Cells were lysed using CelLytic™ M Cell Lysis Reagent (Sigma-Aldrich) supplemented with Halt protease and phosphatase inhibitor cocktail (Thermo Fisher Scientific). Total protein concentration was determined using Bradford assay (Bio-Rad) and equal amounts of total protein were then boiled at 95°C for 5 min with 4× Laemmli Sample Buffer (Bio-Rad), and then separated on SDS-PAGE gels. Separated proteins were then transferred onto PVDF membranes (Santa Cruz Biotechnology) following standard methods. After blocking with 5% nonfat dry milk in TBS with 0.1% Tween 20 (TBST) for 1 h at room temperature, membranes were incubated overnight with the following primary antibodies at 4°C: beta-actin (Abcam; ab6276), hTERT (Abcam; ab94523) and N-Cadherin (Abcam; ab76011). Membranes were washed in PBST three times prior to a 1 h incubation with Goat Anti-Mouse IgG (H+L)-HRP Conjugate or Goat Anti-Rabbit IgG (H+L)-HRP Conjugate secondary antibodies (Bio-Rad) at a 1:3000 dilution in 5% PBST-milk. After three washes in TBST, the membranes were developed with enhanced chemiluminescence detection reagent, ECL blotting substrate (Bio-Rad). The signal on membranes was visualized using ChemiDoc™ MP Imaging System (Bio-Rad).

### Chemotherapy sensitivity assay

Cells were plated in a 12-well plate at a density of 4×10^5^ cells/well. Cells were then treated with cisplatin at concentrations of (5, 10, 15, 20 and 25 µM) or Doxorubicin (2, 6 and 10 nM). After incubation for 24 h, the viability and apoptosis induced by anticancer regimens were analyzed by flow cytometry using an Annexin-V-FITC and propidium iodide (PI) apoptosis detection kit (Miltenyi Biotec Inc., Auburn, CA, USA) as per the manufacturer's protocol. Experiments were each performed three times in triplicate.

### Confocal fluorescence microscopy immunostaining

Cells were seeded on coverslips, fixed with 4% paraformaldehyde, permeabilized with 0.1% Triton X-100, and blocked with 4% BSA. Cells were then stained with hTERT antibody (Abcam), E-Cadherin antibody (Cell Signaling Technology), N-Cadherin antibody (Abcam), Snail+Slug antibody (Abcam), ALDH1A1 antibody (Pierce Antibodies) and β-Catenin antibody (Cell Signaling Technology). The primary antibodies were detected by using an appropriate Alexa Fluor^®^ secondary antibodies (Molecular Probes) and counterstained with Hoechst 33342 (Molecular Probes) to visualize the cell nuclei. Cells were imaged under a 60X objective with Nikon A1R inverted laser scanning confocal microscope (Nikon Microsystems, Massy, France).

### Real-time qPCR

RNA was extracted using the PureLink^®^ RNA Mini Kit (Life Technologies) according to the manufacturer's instructions and treated with DNAse I (Sigma-Aldrich). The cDNA was synthesized by using the iScript™ cDNA Synthesis Kit (Bio-Rad) and quantitative Real-Time PCR assay was performed using SsoAdvanced™ Universal SYBR^®^ Green Supermix (Bio-Rad) on the QuantStudio™ 12K Flex Real-Time PCR System (Applied Biosystems, Foster City, CA, USA). The sequences of the used primers are indicated in Table S1. The relative gene expression was calculated using the comparative threshold (2^ΔΔCT^) method and the data were normalized to β-actin gene expression. Each experiment was performed twice and each reaction was performed in triplicates.

### Tumorsphere formation assay

The tumorsphere formation assay was performed as previously described with slight modifications (Dontu et al., 2003). Single-cell suspensions were plated in ultra-low attachment flasks in DMEM-F12 with 2% B27 supplement (Life Technologies), 20 ng/ml epidermal growth factor (EGF, Life Technologies), 20 ng/ml bFGF (Life Technologies), 10 µg/ml insulin and 10 µg/ml hydrocortisone. Tumorspheres were cultured for 8 days, then the cells collected from non-adherent cultures were quantified with a Bio-Rad TC20™ Automated Cell Counter (sizing range of 20–336 µm). Experiments were each performed three times in triplicate.

### *In vitro* vasculogenesis tube formation assay

As previously described (El-Badawy et al., 2016), cells were seeded in 24-well plates pre-coated for 30 min at 37°C with Geltrex^®^ LDEV-Free Reduced Growth Factor Basement Membrane Matrix (Invitrogen) at the density of 1.5×10^6^ in 250 μl of large vessel endothelial-supplemented Medium 200 (Gibco) and incubated overnight at 37°C in a humidified atmosphere of 5% CO_2_. After 16 h, cells were stained with 2 μg/ml of Calcein, AM (Molecular Probes) for 30 min and then imaged using a Leica DMi8 inverted fluorescent microscope (Leica Microsystems, Wetzlar, Germany).

### Stress induced injury and MTT assay

For inducing oxidative stress, cells were cultured in six-well plates and H_2_O_2_ treatment was carried out 24 h after seeding in media containing 600μM H_2_O_2_ for 48 h. For serum starvation, cells were cultured in DMEM supplemented in 1% FBS for 48 h. Following the treatments, the MTT reagent 3-(4,5-dimethylthiazol-2-yl)-2,5-diphenyltetrazolium bromide (Life Technologies) was added to each well of cells at a concentration of 5 mg/ml and incubated in a humidified 5% CO_2_ incubator at 37°C for 3 h. The formazan salts were dissolved with DMSO for 15 min and the optical density was measured at 570 nm with reference to 630 nm by using a FLUOstar Omega-microplate reader (BMG Labtech, Cary, NC, USA).

### Migration assay

For the migration assay, a confluent monolayer of cells was subjected to serum starvation for 16 h., then scratched with a pipette tip, washed with PBS, and incubated in culture medium supplemented with 10% FBS. The cultures were photographed using phase-contrast microscopy at 0, 12, 24 and 48 h. All experiments were performed in triplicate.

### Colony formation assay

Cells were seeded in a six-well plate at a density of 200 cells/well. After 10 days, colonies were fixed, stained with 1% Giemsa Stain in methanol and only colonies consisting of more than 50 cells were counted.

### Statistical analysis

All the data are presented as mean±standard deviation (SD). An unpaired two-tailed Student *t*-test was used to calculate *P*-values. Statistical significance was identified at *P*<0.05.

## Supplementary Material

Supplementary information
