# Supplementary material for: Telomerase reverse transcriptase coordinates with the epithelial-to-mesenchymal transition through a feedback loop to define properties of breast cancer stem cells
Source: Biol Open. 2018 Jun 15;7(7):bio034181. doi: 10.1242/bio.034181 (PMC6078341; doi:10.1242/bio.034181)
Supplement: Supplementary information [file biolopen-7-034181-s1.pdf]

Supplementary Table S1: Sequence of Primers used

Gene Name	Primer Sequence
Human β-actin Forward	AGAGCTACGAGCTGCCTGAC
Human β-actin Reverse	AGCACTGTGTTGGCGTACAG
Human hTERT Forward	CGGAAGAGTGTCTGGAGCAA
Human hTERT Reverse	GGATGAAGCGGAGTCTGGA
Human CDK4 Forward	TCGAAAGCCTCTCTTCTGTG
Human CDK4 Reverse	TACATCTCGAGGCCAGTCAT
Human CDK6 Forward	GCTGGTAACTCCTTCCCCAG
Human CDK6 Reverse	GTCCAGAATCATTGCACCTGAG
Human TP53 Forward	GTTCCGAGAGCTGAATGAGG
Human TP53 Reverse	TTATGGCGGGAGGTAGACTG
Human E2F3 Forward	GAGACTGAAACACACAGTCC
Human E2F3 Reverse	CCTGAGTTGGTTGAAGCC
Human SMAD7 Forward	AGAAGGTGCGGAGCAAAAT
Human SMAD7 Reverse	GTGTGGCGGACTTGATGA
Human HER2 Forward	ATCTGCCTGACATCCACG
Human HER2 Reverse	GCAATCTGCATACACCAGTTC

Biology Open • Supplementary information

Human KRAS Forward	TGTTCACAAAGGTTTTGTCTCC
Human KRAS Reverse	CCTTATAATAGTTTCCATTGCCTTG
Human ALDH1A1 Forward	CGCAAGACAGGCTTTTCAG
Human ALDH1A1 Reverse	TGTATAATAGTCGCCCCCTCTC
Human ABCG2 Forward	TTTCCAAGCGTTCATTCAAAAA
Human ABCG2 Reverse	TACGACTGTGACAATGATCTGAGC
Human CD90 Forward	TCAGGAAATGGCTTTTCCCA
Human CD90 Reverse	TCCTCAATGAGATGCCATAAGCT
Human Nestin Forward	AGCGTTGGAACAGAGGTTGGA
Human Nestin Reverse	TGTTTCCTCCCACCCTGTGTC
Human EpCAM Forward	CGCAGCTCAGGAAGAATGTG
Human EpCAM Reverse	TGAAGTACACTGGCATTGACG
Human Oct4 Forward	TGTACTCCTCGGTCCCTTTC
Human Oct4 Reverse	TCCAGGTTTTCTTTCCCTAGC
Human Sox2 Forward	GCTAGTCTCCAAGCGACGAA
Human Sox2 Reverse	GCAAGAAGCCTCTCCTTGAA
Human Nanog Forward	CAGTCTGGACACTGGCTGAA
Human Nanog Reverse	CTCGCTGATTAGGCTCCAAC
Human Bcl-2 Forward	GGATAACGGAGGCTGGGATG

Biology Open • Supplementary information

Human Bcl-2 Reverse	TGACTTCACTTGTGGCCCAG
Human Bax Forward	CAAACTGGTGCTCAAGGCCC
Human Bax Reverse	GAGACAGGGACATCAGTCGC
Human PARP Forward	AGCGTGTTTCTAGGTCGTGG
Human PARP Reverse	CATCAAACATGGGCGACTGC
Human N-Cadherin Forward	GGTGGAGGAGAAGAAGACCAG
Human N-Cadherin Reverse	GGCATCAGGCTCCACAGT
Human SNAIL Forward	ACCACTATGCCGCGCTCTT
Human SNAIL Reverse	GGTCGTAGGGCTGCTGGAA
Human Slug Forward	TGTTGCAGTGAGGGCAAGAA
Human Slug Reverse	GACCCTGGTTGCTTCAAGGA
Human ZEB1 Forward	GATGATGAATGCGAGTCAGATGC
Human ZEB1 Reverse	CTGGTCCTCTTCAGGTGCC
Human Twist1 Forward	CGGGAGTCCGCAGTCTTA
Human Twist1 Reverse	GCTTGAGGGTCTGAATCTTG
Human Vimentin Forward	TGTCCAAATCGATGTGGATGTTTC
Human Vimentin Reverse	TTGTACCATTCTTCTGCCTCCTG

Biology Open • Supplementary information

Human Desmoplakin Forward	CAGTGGTGTCAGCGATGATGT
Human Desmoplakin Reverse	TGACGCTGGATATGGTGGAA

Biology Open • Supplementary information
